# Trends in non-fatal agricultural injuries requiring trauma care

**DOI:** 10.1186/s40621-015-0062-3

**Published:** 2015-12-04

**Authors:** Celestin Missikpode, Corinne Peek-Asa, Tracy Young, Amanda Swanton, Kathy Leinenkugel, James Torner

**Affiliations:** 1 Injury Prevention Research Center, University of Iowa, 2190 WL, Iowa City, IA 52242 USA; 2 Department of Epidemiology, College of Public Health, University of Iowa, 145 N. Riverside Drive, Iowa City, IA 52242 USA; 3 Department of Occupational and Environmental Health, College of Public Health, University of Iowa, 145 N. Riverside Drive, 100 CPHB, S143, Iowa City, IA 52242 USA; 4 Iowa Department of Public Health, 321 E. 12th Street, Des Moines, IA 50319 USA

**Keywords:** Agriculture, Injury, Non-fatal, Trends

## Abstract

**Background:**

Efforts to control agricultural injuries have been underway for years. Yet, very little is known about their trends over time. We examined trends in non-fatal agricultural injuries through analyzing injuries reported in a state trauma registry.

**Methods:**

Using Iowa Trauma Registry data collected by the Iowa Department of Public Health, we examined trends in non-fatal agricultural injuries reported by acute care hospitals accredited as Level I, II, and III Trauma Care Facilities from 2005 to 2013. Rate ratios and corresponding 95 % confidence intervals were used to examine the burden of non-fatal agricultural injuries across this period. Negative binomial regression was used to calculate the average annual change in agricultural injury rates over time. Joinpoint regression analysis was used to examine the average annual change in the number of injuries over time.

**Results:**

Between 2005 and 2013, a total of 1238 agricultural injuries were reported to the trauma registry by Level I, II and III trauma facilities. From 2005 to 2013, the rate of agricultural injuries per 100,000 hired workers, ranchers, and farm operators increased by 11 % for every unit increase in year and had nearly tripled over this time period. From 2005 to 2008 there was a significant annual increase of 31.74 % in the number of agricultural injuries whereas from 2008 to 2013 there was a non-significant annual increase of 3.70 %. The number of moderate and severe/critical injuries increased steadily and significantly over the study period, with annual percent increases of 13 and 20 %, respectively.

**Conclusion:**

Non-fatal agricultural injuries are rising, although the documented increases could be influenced in some part by treatment patterns in the trauma system, reporting bias or increases in farm work exposure. However, these issues do not likely account for all of the increase found, and this calls for an increase in priority of agricultural safety programs. Since the majority of research involves fatal injuries, information about non-fatal injuries may help inform new intervention approaches.

## Background

With an ever-increasing world population, the future of agriculture will require increased food production while there is a concomitant reduction in the global rural population. Although agriculture is an important economic component of almost every country, farming ranks among the most dangerous occupations worldwide and is associated with a relatively high risk of injury, disability, and death. The International Labor Organization estimates that more than 170,000 deaths are associated with agricultural work every year (International Labor Organization 2009). According to the US Bureau of Labor Statistics (BLS), there were a total of 422 workers who died in the agricultural production sector in 2007, yielding an annual fatality rate of 24.2 deaths per 100,000 workers (US Bureau of Labor Statistics 2007). Despite control efforts, there is still a lack of progress in substantially reducing fatality rates, which remained at 20.3 per 100,000 workers in 2013 (US Bureau of Labor Statistics 2013). In general, injuries associated with the agricultural industry are severe, and often associated with significant soft-tissue damage, neurovascular injury, multilevel fractures, and amputations (Yaffe & Kaplan 2014). The risk of dying from farming is more than five times higher than the risk for all other occupations combined (Toscano 1997). Numerous studies have linked agricultural injuries to machinery including tractors, power take-off devices, grain augers, hay balers, and combine harvesters (Toscano 1997; Pickett et al. 2001; Hartling et al. 1998). In one tertiary hospital, 90 % of patients admitted for agricultural injuries required surgical intervention, and the length of hospital stay averaged 5 days (Jawa et al. 2013). These injuries are associated with high risk of disability. Studies have reported up to 135 days of disability with a high prevalence of permanent disability (Hansen & Carstensen 1999; Campbell et al. 1979; Athanasiov et al. 2006). The economic cost associated with agricultural-related injuries is substantial. For example, in 1992, the cost of medical care for management of agricultural injuries in the United States was $4.57 billion (Leigh et al. 2001).

Given the high fatality and disability associated with agricultural injuries, and the tremendous medical care cost, increased focus on prevention is an effective means to address this health problem and reduce healthcare costs. Preventing agricultural injuries necessarily involves surveillance in order to identify the burden and associated risk factors. Surveillance of fatal agricultural injuries in the United States is routinely conducted through the Census of Fatal Occupational Injuries (CFOI) program. However, less information is available about non-fatal injuries. Although fatal injury rates have not been decreasing, the trends of non-fatal agricultural injuries over time are not clear.

The Iowa Trauma System, established through the Iowa Trauma Act of 1995 and implemented in January 1, 2001, monitors trauma care throughout the state. The Iowa Trauma Registry is the surveillance component of the Trauma System, and a unique feature of this statewide trauma registry is the specific identification of agricultural-related injuries. The purpose of the present study was to characterize patterns in non-fatal agricultural injuries through analyzing farm surveillance data. This analysis will inform surveillance, research, and intervention to identify changes in burden and characteristics of agricultural injuries.

## Methods

### Data collection

The Iowa Trauma Registry is a data repository managed by the Iowa Department of Public Health (IDPH) and is the surveillance component used for quality improvement of the Iowa Trauma System. Iowa acute care hospitals are certified at one the four levels of trauma care following guidelines from the American College of Surgeons [https://www.facs.org/]. The Iowa Trauma System is comprised of 118 acute care hospitals or medical centers in Iowa, including 2 Level I, 4 Level II, 19 Level III, and 93 Level IV trauma care facilities. Level I trauma care facilities provide the highest level of specialty care, as well as providing leadership in education, research, and system planning. Level II trauma care facilities are capable of providing definitive trauma care regardless of the severity of injury, and provides 24-h availability of all essential specialties, personnel, and equipment yet they don’t have the same research and education requirements as Level I centers. Level III trauma care facilities have the resources to provide stabilization for all trauma patients and may provide surgical and/or critical care when appropriate. Level IV trauma care facilities provide initial evaluation, stabilization, diagnostic capabilities, transfer to a higher level of care when appropriate, and may also provide surgical care when appropriate.

Acute care trauma care facilities designated as Level I, II, or III are required through the State Trauma Act to report information about trauma cases to the Iowa Trauma Registry. Reporting by Level IV trauma care facilities was voluntary during the years represented in this study, with only some of their reports available in the data set. Reporting is required for patients who had at least one injury ICD-9 diagnosis code between 800.00 and 959.9 and met one of two conditions: they were admitted to a Level I –III trauma care facility, transferred to a Level I –III trauma care facility, or the patient died in the trauma care facility; or, the trauma care facility team was activated. Because of concerns about incomplete reporting into the data system by Level IV facilities, we only included data reported by Level I, II, and III trauma facilities in the current analysis.

For the registry, abstracted data from medical records are submitted by trauma nurses/registrars at each trauma care facility within 90 days of the injury and entered into the Iowa Department of Public Health registry. The University of Iowa Injury Prevention Research Center access to the data is managed through a Data Sharing Agreement (DSA 268).

For this study, injuries in the Iowa Trauma Registry data from 2005 to 2013 that were agricultural work-related were included. The Iowa Trauma Registry has separate indicators for farm-related and work-related, and this study sample included Iowa Trauma Registry entries for which both of these indicators were positive. Based on the Iowa Trauma Registry Data Dictionary, a farm-related injury was defined as a non-household injury incurred on the farm by any farmer, farm worker, farm family member, or other individual, or any non-farm injury incurred by a farmer, farm worker, or farm family member in the course of handling, producing, processing, transporting, or warehousing farm commodities. Farming includes crop and animal production. Injuries incurred by farmers or non-farmers who are on farm environs for purposes unrelated to farming activity (e.g., visiting, hunting, swimming, and other recreational activities) and farmhouse or home premises of the farm are excluded. Work was defined as an activity that is conducted for monetary or non-monetary payment. Of the 113,663 patients in the trauma registry, there were 1903 (1.7 %) and 1688 (1.5 %) that had unknown, missing, or not applicable responses for the farm-related and work-related fields, respectively. These were excluded because we could not determine if the patients met case criteria. A total of 1238 patients that met the criteria for being a farm and/or work-related injury were identified. The number of patients seen at each level of trauma care facilities included: 512 Level I (41.4 %); 337 Level II (27.2 %), and 389 Level III (31.4 %).

Information extracted from the trauma registry included: demographics of trauma patients, diagnostic code information, individual hospital identifiers, level of trauma care facility, date of injury, mechanism of injury, type of injury, and severity of injury measured by injury severity score (ISS). The ISS is an established anatomical score that measures the overall severity of injury in trauma patients (Baker et al. 1974; Semmlow & Cone 1976; Maurer & Morris 2004). The ISS is derived from the Abbreviated Injury Scale (AIS), which is a severity scoring method that classifies each injury by body region based on its relative severity on a six point ordinal scale: 1 = minor, 2 = moderate, 3 = serious, 4 = severe, 5 = critical, 6 = maximal (untreatable). Only the highest AIS score from each of the six body regions (head, face, chest, abdomen, extremities, and external) is considered for ISS calculation. ISS is calculated by squaring and summing together the 3 most severely injured body regions. The maximum ISS score is automatically assigned if any of the injuries is scored 6 on AIS scale. The ISS score ranges from 0 to 75 and a score greater than 15 indicates a major trauma.

### Denominator data

We derived the numbers of hired workers, ranchers, and farm operators from the Census of Agriculture conducted by the National Agricultural Statistics Service (NASS). The NASS collects data on farm operations, hired workers, ranchers, and farm operators at the national, State, and county level every 5 years. The Census of Agriculture provides the most stable and accurate estimates of exposure to farming and thus provide stable estimates over time, which was the main aim of this analysis. Two Censuses of Agriculture surveys were conducted over our study period (in 2007 and 2012). According to these censuses, there were 207,992 and 211,373 hired workers, ranchers, and farm operators in Iowa in 2007 and 2012 respectively (USDA Census of Agriculture 2015). To estimate the number of hired workers, ranchers, and farm operators per year, we determined the average annual change based on the two available time periods (2007 and 2012). We found that the number of hired workers, ranchers, and farm operators increased by 676 for every unit increase in year. We applied this annual change to each study year.

### Statistical analysis

**Fig. 1:**
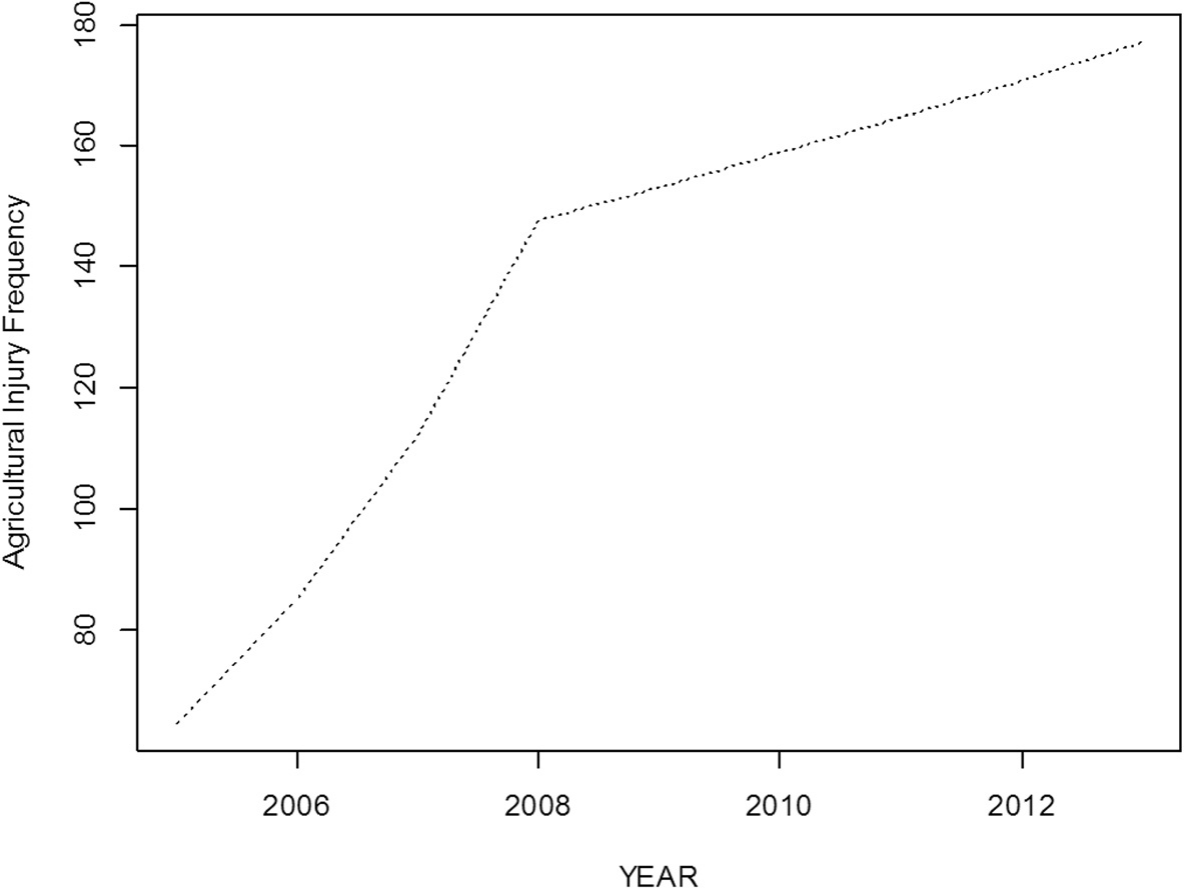
Trends in non-fatal agricultural injury requiring trauma care

**Fig. 2:**
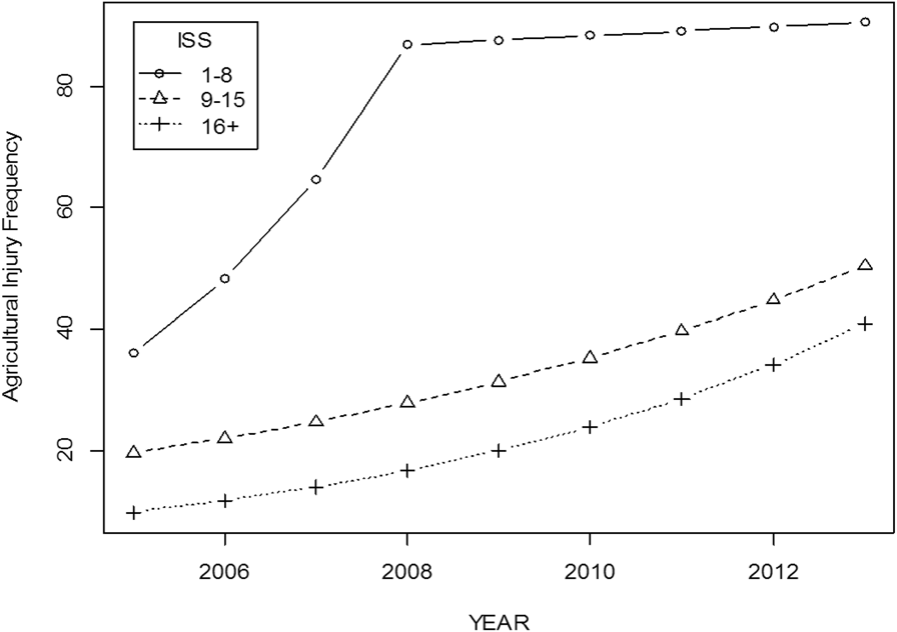
Trends by injury severity categories

Injury rates for Level I, II, and III trauma facilities were calculated as the number of agricultural injuries divided by the annual estimated number of hired workers, ranchers, and farm operators for the years 2005–2013. We further calculated rate ratios and 95 % confidence intervals using the year 2005 as the reference. To model the injury rate as a function of time (year), we first fitted a Poisson regression in SAS software, which suggested evidence of overdispersion. We then used negative binomial regression, which was a better fit with our data. The number and proportion of injuries were examined for each year by age, gender, mechanism of injury, and type of injury. We explored trends in agricultural injuries and by injury severity. To examine trends in agricultural injuries according to severity, we stratified the data using injury severity score (ISS). We separately explored trends in minor injuries (ISS 1–8), moderate injuries (ISS 9–15), and severe/critical injuries (ISS 16 +). The joinpoint regression program software, V.4.0.1, was used to test the statistical significance of the different trends. We used the log-linear model which computes the Annual Percent Change (APC) [http://surveillance.cancer.gov/joinpoint]. Because we saw a significant increase in moderate and severe/critical injuries over the entire study period, we further fitted a trend line (0 joinpoint) to the data by age group, gender, mechanism and type of injury using linear models to further characterize factors behind these observed trends. The results of the linear models are presented as intercept, slope and its p-value. All analyses were done in joinpoint regression program software and SAS. Graphs of Figs. 1 and 2 were generated in R.

## Results

**Fig. 3:**
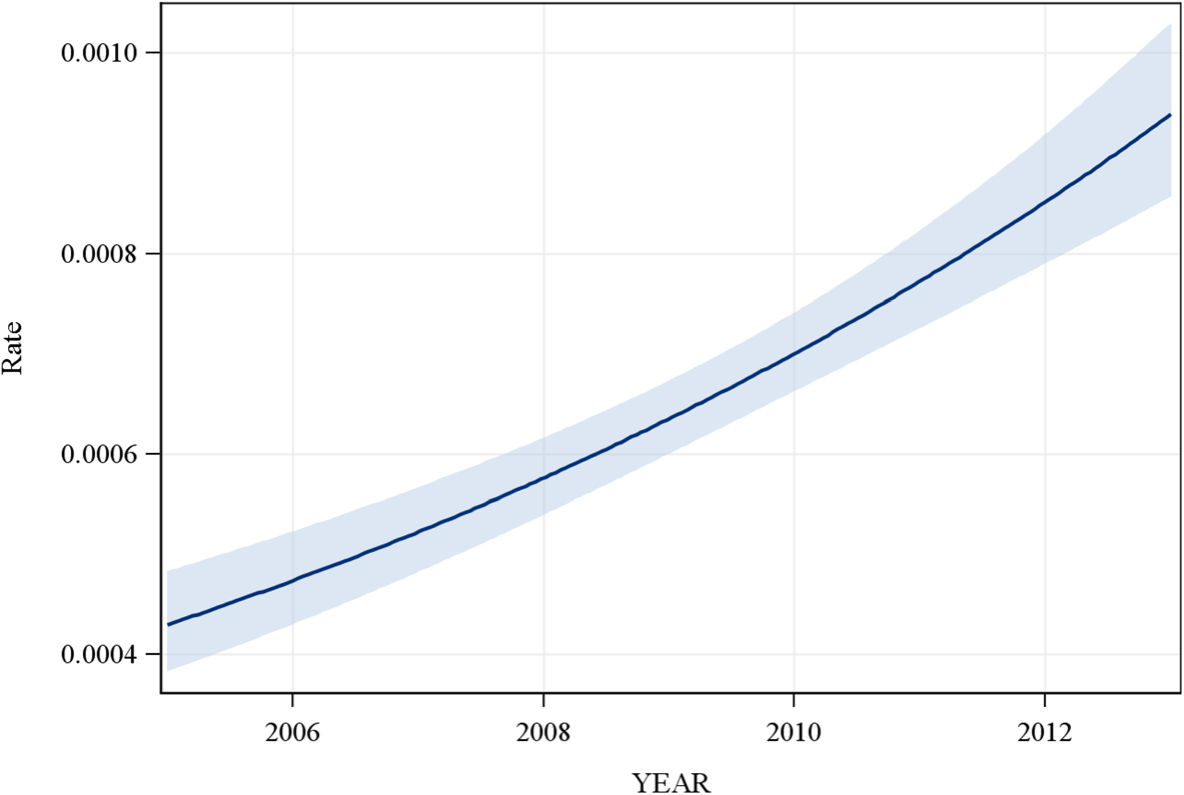
Plot of the fitted injury rate model with 95 % confidence limits

Between 2005 and 2013, a total of 1238 agricultural injuries were reported to the trauma registry by Level I, II and III trauma facilities. Table 1 provides the distribution of these injuries over time as well as the injury rates per 100,000 hired workers, ranchers, and farm operators per year. A steep increase in the number of injuries was observed from 2005 to 2008, followed by a steady increase through 2013 (Fig. 1). Overall, from 2005 to 2008 there was a significant annual increase of 31.74 % in the number of agricultural injuries whereas from 2008 to 2013 there was a non-significant annual increase of 3.70 %. Moderate and severe/critical injuries increased significantly and steadily over the study period, with annual percent increases of about 13 and 20 %, respectively (Fig. 2). The rate of agricultural injury per 100,000 hired workers, ranchers, and farm operators dramatically increased over the entire study period. Compared to 2005, this rate is nearly 3 times the rate in 2013 (Rate ratio = 2.72; 95%CI 2.04–3.63) (Table 1). The injury rate model showed that this rate increases by 11 % for every unit increase in year as depicted by Fig. 3.

**Table 1:** Injury rates, rate ratios and 95 % confidence intervals, and injury rate model estimate

Year	Number of injuriesN (%)	Number of hired workers, ranchers, and farm operators	Injury rate per 100,000 hired workers, ranchers, and farm operators (95 % CI)	Rate ratios (95 % CI)
2005	63 (5.09)	206,640	30.49 (22.72, 38.26)	1.00 (Ref.)
2006	95 (7.67)	207,316	45.82 (36.37, 55.28)	1.50 (1.09, 2.07)
2007	97 (7.84)	207,992	46.64 (37.12, 56.16)	1.53 (1.11, 2.10)
2008	148 (11.95)	208,669	70.93 (59.26, 82.59)	2.33 (1.73, 3.12)
2009	174 (14.05)	209,345	83.12 (70.53, 95.70)	2.73 (2.04, 3.64)
2010	149 (12.04)	210,021	70.95 (59.32, 82.57)	2.33 (1.73, 3.12)
2011	160 (12.92)	210,697	75.94 (63.94, 87.94)	2.49 (1.86, 3.33)
2012	176 (14.22)	211,373	83.27 (70.73, 95.80)	2.73 (2.05, 3.64)
2013	176 (14.22)	212,050	83.00 (70.51, 95.49)	2.72 (2.04, 3.63)
All	1238 (100)			

Regression estimate β for injury rate model (*e* ^*β*^)

β= 0.1065 (*e* ^*β*^ =1.11); *p*-value <0.0001

Table 2 shows the number and proportion of injuries for each year by age, gender, mechanism of injury, and type of injury, as well as the results of their linear models. Each year, males accounted for the vast majority of agricultural injuries. Additionally, each year, all agricultural injuries were experienced more often by those aged 21–64 years than the age groups 65 years or older and 0–20 years. Machinery and falls remained the leading mechanisms of injury over the study period. Agricultural injuries often resulted in significant trauma. Fracture was the leading type of injury every year.

**Table 2:** Number of injuries by age groups, mechanism and type, and regression estimates

	2005 N (%)	2006 N (%)	2007 N (%)	2008 N (%)	2009 N (%)	2010 N (%)	2011 N (%)	2012 N (%)	2013 N (%)	Intercept	Slope	*P*-value for slope
Age^a^												
0 – 20	7 (11.1)	8 (8.4)	NR^b^	NR	9 (5.3)	12 (8.1)	7 (4.4)	15 (8.5)	9 (5.1)	4.94	0.70	0.1132
21 – 64	41 (65.1)	67 (70.5)	74 (76.3)	117 (79.0)	124 (72.9)	100 (67.1)	129 (80.6)	130 (73.9)	114 (64.8)	51.72	9.57	0.0061
65 +	15 (23.8)	20 (21.1)	19 (19.6)	26 (17.6)	37 (21.8)	37 (24.8)	24 (15.0)	31 (17.6)	53 (30.1)	11.94	3.43	0.0104
Gender^a^												
Male	49 (77.8)	86 (90.5)	94 (96.9)	134 (91.2)	156 (89.7)	139 (93.3)	149 (93.7)	168 (95.5)	165 (93.8)	57.92	13.75	0.0005
Female	14 (22.2)	NR	NR	13 (8.8)	18 (10.3)	10 (6.7)	10 (6.3)	8 (4.5)	11 (6.2)	11.00	−0.07	0.9112
Mechanism^a^												
Machinery	16 (26.2)	19 (20.4)	20 (22.7)	42 (29.0)	43 (25.3)	35 (24.0)	37 (23.1)	34 (19.3)	32 (18.2)	19.56	2.27	0.0784
Fall	15 (24.6)	17 (18.3)	18 (20.5)	31 (21.4)	41 (24.1)	27 (18.5)	39 (24.4)	36 (20.5)	28 (15.9)	15.75	2.45	0.0392
Transportations	12 (19.7)	16 (17.2)	15 (17.1)	19 (13.1)	25 (14.7)	32 (21.9)	19 (11.9)	28 (15.9)	30 (17.1)	11.03	2.15	0.0067
Struck by/against	NR	NR	9 (10.2)	16 (11.0)	19 (11.2)	13 (8.9)	13 (8.1)	27 (15.3)	22 (12.5)	6.64	1.78	0.0313
Natural/ Environment	NR	NR	6 (6.8)	17 (11.7)	9 (5.3)	13 (8.9)	20 (12.5)	22 (12.5)	32 (18.2)	−0.19	2.95	0.0011
Cut/pierce/fire/burn/assault/suicide/firearm/undetermined	6 (9.8)	8 (8.6)	12 (13.6)	8 (5.5)	19 (11.2)	14 (9.6)	20 (12.5)	21 (11.9)	10 (5.7)	6.69	1.28	0.0762
Other specified/unspecified	NR	NR	8 (9.1)	12 (8.3)	14 (8.2)	12 (8.2)	12 (7.5)	8 (4.6)	22 (12.5)	4.36	1.35	0.0332
Type^a^												
Fracture	26 (43.3)	38 (41.8)	26 (29.2)	57 (39.0)	62 (40.5)	69 (47.6)	65 (41.4)	74 (44.3)	82 (47.4)	20.28	7.03	0.0002
Head Injury/SCI/Nerves^c^	12 (20.0)	8 (8.8)	16 (18.0)	19 (13.0)	19 (12.4)	13 (9.0)	23 (14.7)	30 (18.0)	19 (11.0)	9.17	1.70	0.0292
Amputation/Crushing/Other Injury	9 (15.0)	23 (25.3)	20 (22.5)	21 (14.4)	30 (19.6)	22 (15.2)	25 (15.9)	16 (9.6)	18 (10.4)	18.28	0.43	0.6036
Open Wound	6 (10.0)	8 (8.8)	13 (14.6)	25 (17.1)	16 (10.5)	15 (10.3)	16 (10.2)	24 (14.4)	14 (8.1)	8.89	1.27	0.1244
Internal organ/BV^c^	NR	NR	NR	10 (6.9)	7 (4.6)	9 (6.2)	7 (4.5)	11 (6.6)	15 (8.7)	1.61	1.23	0.0047
Burn	NR	NR	NR	6 (4.1)	8 (5.2)	11 (7.6)	12 (7.6)	6 (3.6)	11 (6.4)	2.58	0.95	0.0123
Dislocation/Sprain	NR	NR	6 (6.7)	8 (5.5)	11 (7.2)	6 (4.1)	9 (5.7)	6 (3.6)	14 (8.1)	2.50	0.97	0.0264

Age group 0 – 20: = 9 (2007/2008); Female: *N* = 12 (2006/2007); Struck by/against: *N* = 21 (2005/2006); Natural/environment: *N* = 12 (2005/2006); Other specified/unspecified: *N* = 12 (2005/2006); Internal organ/BV: *N* = 11 (2005/2006/2007); Burn: *N* = 12 (2005/2006/2007); Dislocation/sprain: *N* = 6 (2005/2006)

^a^Missing values on age (*N* = 4), gender (*N* = 2), mechanism (*N* = 23), type (*N* = 57)

^b^NR – unreported. Per our data use agreement, cell counts of 5 or less cannot be disclosed; thus they are aggregated with adjacent counts as shown below

^*c*^ *Abbreviations*: *SCI*= spinal cord injury, *BV*= blood vessel

The number of agricultural injuries significantly increased over time among males and in the age groups 21–64 and 65+ (Table 2). Machinery was consistently the leading mechanism of injury, and its increase over the study period was marginally significant (*p*-value = 0.08). Natural/environmental mechanisms (excessive heat; excessive cold; high and low air pressure and changes in air pressure; travel and motion; hunger thirst exposure and neglect; venomous animals and plants as the cause of poisoning and toxic reactions; other injury caused by animals such as dog bite, rat bite, bite of nonvenomous snakes; lightning; cataclysmic storms and floods resulting from storms; cataclysmic earth surface movements and eruptions) yielded the most significant increased number of injuries (*p*-value = 0.001) followed by transportation (*p*-value = 0.007). Other important mechanisms attributable to the increase in agricultural injuries over time were falls, struck by/against, other specified/unspecified mechanisms, and fire/burn. The types of injury that significantly increased over time were fracture, head/spinal cord/nerves injuries, internal organ/blood vessel injuries, burn, and dislocation/sprain.

## Discussion

This study found a significant increase in the agricultural injury rate per 100,000 hired workers, ranchers, and farm operators who were treated at a Level I, II, or III trauma facility, which had nearly tripled over an 8 year period, from 30.49 per 100,000 hired workers, ranchers, and farm operators in 2005 to 83.00 in 2013. This trend was largely due to a steep increase in less severe injuries for the first 3 years of the study period, which could be attributable to changes in admission and transfer patterns in the trauma system. However, we further found that moderate and severe/critical injuries increased significantly and steadily over the study period with annual percent changes of 13 and 20 %, respectively. This suggests that the number of injuries occurring in agricultural industry continues to be a concern and warrants further public health attention.

The data utilized represent only the more severe non-fatal agricultural injuries because we excluded data from Level IV (community) trauma facilities, which represent almost 79 % of all trauma facilities in Iowa. With exclusion of Level IV trauma facilities, our rates do not reflect the overall rates of non-fatal agricultural injuries requiring trauma care. However, this exclusion is unlikely to affect measured trends in severe agricultural injuries because trauma Level IV facilities may lack the expertise and resources required for complete management of serious injuries, and routinely transfer moderate to severe cases to upper level trauma sites for definitive care. It remains possible that some level IV centers may have tried to treat few severe injuries because of transfer issues. These few cases, however, are unlikely to influence measured trends in moderate and severe injuries.

We found a decline in the number of agricultural injuries among females while the trend for male injuries has shown a significant increase. This difference in injury trends by sex could be explained by changes in task assignments. Women may have worked in less dangerous tasks over time while men may have continued to engage in more hazardous tasks. Greater safety practiced by women compared to men (Van den Broucke & Colémont 2011) could also be influencing changes in exposure. The non-increase in agricultural injuries over time among individuals 20 years old or younger may partially be attributable to prevention efforts such as the North American Guidelines for Children’s Agricultural Tasks (Lee & Marlenga 2005), which provide factors for parents to consider in task assignment. A significant decline in the number of agricultural injuries among individuals less than 20 years old was observed between1998 to 2006 in another study (Hendricks & Hendricks 2010). The non-decline in youth injury found in the current study may be due to a focus on more severe injuries and the exclusion of facilities accredited as trauma level IV. We found that agricultural injuries among adults and elderly individuals have shown a significant increase over time, which could be the result of the growing segment of the older farming population and their vulnerability due to the physiological changes that can accompany aging, such as the occurrence of age-related chronic diseases. Some older adults might begin a career in farming after retirement from other fields and others might remain longer in the farming because they have strong passion for the farm work. An increasing average age for farmers and ranchers has been reported by the USDA National Agricultural Statistics Service showing that the mean age of principal farm operators has increased from 50 years in 1978 to 54 years in 1997, 57 years in 2007, and 58 years in 2012 with 33 % of the nation’s principal operators aged 65 years or older (USDA Census of agriculture 2012).

It remains possible that when exposure time is considered, the risk patterns over time for women, children and young individuals may be different, as their risk per exposure time may be higher. In order to examine changing risk patterns of agricultural injuries, it is necessary to have access to reliable estimates of persons at risk as well as hours worked. Differences in exposure to farm work by age group and gender have been reported. An increase in age is associated with a significant increase in farm work exposure, and females have significantly less farm work exposure compared to males (DeWit et al. 2015). It has been shown that adults have higher rates of injury compared to youths when the denominator of persons at risk is used. However, when hours worked is used in the denominator, youths have higher rates of injury compared to adults (Mongin et al. 2007). Similarly, males have higher injury rates than females according to persons at risk; but when the denominator of hours worked is used, males and females have comparable injury rates (Mongin et al. 2007).

Machinery was responsible for most non-fatal agricultural injuries and showed a marginally significant trend over time. Researchers at the Economic Research Service/USDA have examined changes in farm size in the US and found that larger farms (at least 2000 acres of cropland) had increased from 24.1 % in 2001 to 34.3 % in 2011, and the number of small croplands (1–49 acres) had sharply increased by 100,000 over the same period (MacDonald et al. 2013). This change in farm structure may have resulted in greater exposure to machinery hazards over time. In fact, larger croplands may require longer machinery operational hours and greater need for maintenance. It has been suggested that routine maintenance is associated with greater risk of injury as maintenance tasks often bring farmers into close contact with machinery hazards (e.g. cutting blades) (Narasimhan et al. 2010; Rasmussen et al. 2000; Ingram 2003).

This study shows that non-fatal transportation-related farm injuries significantly increased over the entire study period. Transportation, tractor rollover in particular, has long been identified as a leading cause of death for farmers, but less is known about non-fatal transportation injuries (Murphy et al. 2010). The shift of acreage to larger farms and consolidation of grain storage facilities may lead to increased need for roadway transport for farm equipment, increasing injury rates through increased roadway exposure. Increases in rural roadway traffic may also lead to increased risk for farm equipment crashes (Peek-Asa et al. 2007; Harland et al. 2014).

Despite control efforts to reduce both the number of fatal and non-fatal tractor-related injuries (Myers et al. 2005), this study found a marginally significant increase in machinery injury and a significant increase in transportation-related injury over the study period. A statistically insignificant increasing tractor-related fatality in the region of Midwest was similarly observed between 1992 and 2007 (Myers & Hendricks 2010). This implies that the considerable prevention efforts including worker education/training programs and activities, rollover protective structure (ROPS) design and engineering applications (Myers et al. 2005) have not reached yet their targets and this area warrants further attention.

Falls are also an important cause of non-fatal agricultural injury. This study found that fall-related injuries tied with machinery-related injuries as the leading cause every year from 2005 to 2013. Fall-related injuries significantly increased over time, perhaps associated with the increasing number of older farmers and ranchers (USDA Census of agriculture 2012) and the high risk of falls associated with aging. The high number of falls is consistent with previous studies. The US Bureau of Labor Statistics reported that falls accounted for 18 % of all nonfatal injuries and illnesses in 2000 among workers employed in agriculture (U.S. Department of Labor 2002). Similar findings reported by the National Traumatic Injury Surveillance Project showed that falls accounted for 21 % of non-fatal agricultural injuries in the United States in 1995 (Myers 1995). A review conducted by McCurdy and Carroll reported that a quarter of a similar agricultural injuries were from falls (McCurdy & Carroll 2000).

Fractures were the most common injury diagnosis in this sample, and fractures increased over the study period. Transportation and machinery are common mechanisms leading to fractures, which reflects the significant occupational hazards associated with farming. Such mechanisms often involve high energy transfer which is associated with high risk for fracture (Pickett et al. 2001).

Our study has several limitations. We restricted our analysis to data obtained from accredited Level I, II, and III Trauma Care Facilities, thus limiting our ability to examine statewide trends for all non-fatal agricultural injuries requiring trauma care. Our data include only patients who arrive at a hospital and exclude deaths at the scene. Based on these inclusion criteria, our sample represents only patients treated at a Level I, II, or III trauma hospital and not the overall population of agricultural injuries.

Another limitation of this study is our inability to fully characterize trends in non-fatal injuries in the agricultural industry. In fact, adding Level IV data, which are incomplete, still does not provide the overall rate of occurrence of non-fatal agricultural injuries because the hospital data do not capture all agricultural injuries. This contention is supported by an analysis of National Agricultural Workers Survey showing that about 15 % of farmers and ranchers self-administer treatment after injuries and another 9 % do not seek treatment at all (Thierry & Snipes 2015). This may be due to healthcare costs and/or time for seeking treatment especially when workers are able to work despite their injury. Healthcare costs may limit access to care because agriculture laborers during this study period may have been uninsured, ineligible for benefits, or unable to afford health services (Frank et al. 2013).

Additionally, we relied on the designation of agricultural injuries as reported by trauma facilities. While clear data collection protocols were provided to trauma coordinators and data registrars in the trauma facilities, the accuracy of our data was dependent upon the quality of the report submitted by the sites. This study is unable to measure information bias regarding inaccurate or incomplete reporting of a case as truly meeting the criteria of an agricultural injury.

## Conclusions

The current study suggests that non-fatal agricultural injuries requiring Level l- III trauma care are increasing. These data suggest that prevention efforts that target adults and older farmers and ranchers should be prioritized. Further research work should be directed at identifying accommodations and strategies to prevent injury in aging farmers. In addition, more research is needed to identify those factors that have contributed to the increase in farm injuries caused by natural/environmental mechanisms, transportation, struck by/against, and fire/burn mechanisms. Finally, as agricultural and work-related injury variables are integrated into updated data systems, user training to improve data quality will be necessary to allow more complete surveillance of the burden of all agricultural injuries.
